# Improving Co-Amorphous Drug Formulations by the Addition of the Highly Water Soluble Amino Acid, Proline

**DOI:** 10.3390/pharmaceutics6030416

**Published:** 2014-07-14

**Authors:** Katrine Tarp Jensen, Korbinian Löbmann, Thomas Rades, Holger Grohganz

**Affiliations:** Department of Pharmacy, University of Copenhagen, Copenhagen 2100, Denmark; E-Mails: katrine.jensen@sund.ku.dk (K.T.J.); thomas.rades@sund.ku.dk (T.R.); holger.grohganz@sund.ku.dk (H.G.)

**Keywords:** amino acid, co-amorphous, dissolution rate, DSC, FTIR, salt formation, solid state analysis, stability, XRPD

## Abstract

Co-amorphous drug amino acid mixtures were previously shown to be a promising approach to create physically stable amorphous systems with the improved dissolution properties of poorly water-soluble drugs. The aim of this work was to expand the co-amorphous drug amino acid mixture approach by combining the model drug, naproxen (NAP), with an amino acid to physically stabilize the co-amorphous system (tryptophan, TRP, or arginine, ARG) and a second highly soluble amino acid (proline, PRO) for an additional improvement of the dissolution rate. Co-amorphous drug-amino acid blends were prepared by ball milling and investigated for solid state characteristics, stability and the dissolution rate enhancement of NAP. All co-amorphous mixtures were stable at room temperature and 40 °C for a minimum of 84 days. PRO acted as a stabilizer for the co-amorphous system, including NAP–TRP, through enhancing the molecular interactions in the form of hydrogen bonds between all three components in the mixture. A salt formation between the acidic drug, NAP, and the basic amino acid, ARG, was found in co-amorphous NAP–ARG. In comparison to crystalline NAP, binary NAP–TRP and NAP–ARG, it could be shown that the highly soluble amino acid, PRO, improved the dissolution rate of NAP from the ternary co-amorphous systems in combination with either TRP or ARG. In conclusion, both the solubility of the amino acid and potential interactions between the molecules are critical parameters to consider in the development of co-amorphous formulations.

## 1. Introduction

Several approaches have been described for how to overcome the challenges in preparing oral formulations of poorly water-soluble drugs (categorized as Class II and Class IV drugs in the Biopharmaceutics Classification System, BCS) [1]. One of these approaches is the transformation of a crystalline drug into its amorphous form, taking advantage of the improved solubility and, subsequently, potentially higher bioavailability of this form. However, several difficulties are connected with this formulation strategy, of which the low physical stability of the amorphous form is the most critical one, as the molecules in an amorphous form have a tendency to convert back to the more stable and, thus, less water-soluble, crystalline form [2,3]. The most common approach to overcome this stability problem is to incorporate the amorphous drug into a water-soluble, amorphous polymer, creating a glass solution [4]. This formulation approach is characterized by an increased glass transition temperature (T_g_) and by the molecular dispersion of the drug in the polymer [5,6]. However, glass solutions can result in very large bulk volumes in tablets or capsules, due to the low solid state solubility of the drug in the polymer, thus depending on the intended dose of the drug, making this approach sometimes unfeasible for application [7]. The strengths and weaknesses of various amorphous formulation approaches were recently reviewed in several articles [8,9].

Chieng *et al.* prepared systems in which two drugs were able to stabilize each other in the amorphous form, introducing the term “co-amorphous”. Co-amorphous here refers to an amorphous blend consisting only of low molecular weight components in contrast to glass solutions, which contain the drug together with a polymer [10]. These co-amorphous formulations exhibited the improved physical stability of the amorphous form, as well as the improved dissolution rate of the drugs, compared to the amorphous drugs alone [10,11,12]. Several co-amorphous drug-drug combinations were investigated, and the further advantages of these systems are the small bulk volumes of the blends, as well as the possibility of developing new formulations for combination therapy [11,12]. The general production approach for co-amorphous systems so far has been the ball milling of two poorly water-soluble drugs at a molar ratio of 1:1, hereby producing the most stable systems, as the molecules are assumed to interact on the molecular level in pairs via hydrogen bonds (heterodimers) [10,11,12].

In order to develop a more generally applicable approach, drug mixtures with low molecular weight excipients were investigated. Recently, Löbmann *et al.* extended the co-amorphous approach by formulating co-amorphous systems of the poorly water-soluble drugs, indomethacin and carbamazepine, with amino acids [13]. Compared to polymer-based systems, the amount of excipient was reduced, and thus, there is a potential to avoid large excipient bulk volumes. These co-amorphous mixtures were physically stable over a period of at least six months, while pure amorphous drugs showed poor stability (<7 days). However, differences in the suitability of the various amino acids for this formulation approach were found. Tryptophan (TRP) showed a strong potential as an amorphous stabilizer for the poorly water-soluble drugs, indomethacin and carbamazepine, as the formulations were easy to convert into stable co-amorphous powders. The dissolution rate of indomethacin from this co-amorphous powder was highly increased. However, the dissolution rate of carbamazepine was only increased marginally compared to the crystalline form. In contrast, it was not possible to form co-amorphous mixtures of indomethacin and carbamazepine in the presence of the amino acid, tyrosine (TYR). Phenylalanine (PHE) and arginine (ARG) showed intermediate properties for the formation of co-amorphous systems, although the presence of TRP was beneficial for stabilizing the system [14].

It has also been shown that ARG markedly improves the solubility of naproxen (NAP) upon ball milling, due to a reduction in crystallinity [15]. It was suggested that ARG acts as a stabilizer for aromatic substances, observed as interactions between the guanidinium group of the amino acid and the aromatic moiety of alkyl gallates, an approach referred to as the “arginine-assisted solubilization system (AASS)” [16]. This approach was further applied to indomethacin, where the increased solubility of the drug was explained by possible salt formation and aromatic interactions with the ARG molecules [17]. It has further been suggested that the reason for the highly-improved solubility of NAP in combination with ARG is due to salt formation between the two molecules [18,19].

From previous studies on (non-salt forming) drug amino acid mixtures, it became apparent that amino acids either are good stabilizers or improve the dissolution properties of the drug. Therefore, the first aim of this work was to further develop co-amorphous drug amino acid systems using the poorly water soluble drug, NAP, in combination with one amino acid that acts as an amorphous stabilizer for NAP, *i.e.*, TRP, and a second amino acid (proline, PRO) with high water solubility to further improve the dissolution rate of the drug. The selection of stabilizing amino acids was based on an assessment of possible interactions between the drug and the amino acid, as well as previous experience in the formation of stable co-amorphous systems. TRP has so far proven to be a generally good amorphous stabilizer of small molecules, and hydrophobic π–π interactions between the aromatic rings in TRP and NAP have the potential to stabilize the co-amorphous system further. Salt formation between NAP and ARG is expected to contribute to a highly-stable co-amorphous system, as observed for other acid drugs [14]. ARG and TRP both contain functional amino acid groups, which can form hydrogen bonds with the drug molecules and further contribute to a stabilizing effect. The second aim of this study was to investigate the dissolution rate of the drug from a co-amorphous salt (NAP–ARG) and the influence of PRO to further improve the dissolution rate of NAP. Co-amorphous mixtures were prepared at molar ratios of 1:1 or 1:1:1 by vibrational ball milling and analyzed towards their solid state properties, physical stability and dissolution properties. The 1:1 molar ratio between the components in the binary mixtures was previously shown to create the most stable co-amorphous systems [10]. In the system including a third component, the molecules are expected to interact as trimers, similar to the interactions between the drug and the amino acids in the biological receptor [20], favoring a system in a 1:1:1 molar ratio.

## 2. Materials and Methods

### 2.1. Materials

NAP was obtained from Divis Laboratories Ltd. (Florham Park, NJ, USA), and the amino acids, l-TRP, l-ARG, and l-PRO, were purchased from Sigma-Aldrich (St. Louis, MO, USA). All substances were of reagent grade and used as received. Relevant properties of the raw materials are given in Table 1.

**Table 1:** Molar mass, density and solubility of the raw materials. NAP, naproxen; TRP, tryptophan; ARG, arginine; PRO, proline.

Chemical Substance	Molar Mass, M (g/mol)	Density, ρ (g/cm3)	Solubility (mg/mL)
NAP ^1^	230.26	1.265	6
TRP ^2^	204.23	1.303	–
ARG ^2^	174.20	1.325	87.1
PRO ^2^	115.06	1.376	115

^1^ Solubility in phosphate buffer at 37 °C; pH 7.4 [11,21]; ^2^ solubility data in water at 20 °C as from the Material Safety Data Sheet from Sigma-Aldrich.

### 2.2. Methods

#### 2.2.1. Preparation of Amorphous Materials

Co-amorphous mixtures were prepared by ball milling of 1 g of powder at a molar ratio of drug to amino acids of 1:1, and 1:1:1 when including PRO. The samples were milled at a frequency of 30 Hz for 90 min in 25-mL jars containing two 12-mm stainless steel balls in an oscillatory ball mill (Mixer Mill MM400, Retch GmbH & Co., Haan, Germany), placed in a cold environment (+6 °C).

#### 2.2.2. X-ray Powder Diffraction (XRPD)

X-ray powder diffraction (XRPD) was performed using an X’Pert PRO X-ray Diffractometer (PANalytical, Almelo, The Netherlands) using a Cu *K*α radiation source (λ = 1.54187 Å). An acceleration voltage of 45 kV and a current of 40 mA were used. Samples were scanned in reflection mode between 5° and 35° 2θ with a scan speed of 0.067° 2θ/s and a step size of 0.026°. Data were collected and analyzed using the software, X’Pert Data Collector 2.2i (PANalytical B.V., Almelo, The Netherlands).

#### 2.2.3. Differential Scanning Calorimetry (DSC)

Thermograms were collected using a Perking Elmer Diamond differential scanning calorimeter (DSC; PerkinElmer, Shelton, CT, USA) under a 20 mL/min nitrogen gas flow. Temperature and enthalpy calibration was performed using an indium standard. All samples of approximately 6 mg were measured in aluminum pans with pinhole lids, in a temperature interval from −20 to 170 °C and with a heating rate of 10 K/min. The glass transition temperatures (T_g_, midpoint) were calculated as the mean of three independent measurements determined using Perkin Elmer Pyris software (version 7.0.0.0110, PerkinElmer, Shelton, CT, USA).

#### 2.2.4. Theoretical Tg Values (Gordon Taylor Equation)

The theoretical T_g_ for the binary and ternary co-amorphous mixtures was calculated using the Gordon Taylor equation [22]. For the ternary mixtures, a previously published approach using the binary co-amorphous amino acid-amino acid mixture as one component and the drug as the second component was used [13].

The theoretical glass transition temperature was then calculated based on the mean of three measurements of the T_g_ on the amorphous drug, each amino acid in the amorphous form, as well as co-amorphous ARG–PRO and TRP–PRO. The calculation further included the molar masses and densities of the crystalline drug and amino acids listed in Table 1.

#### 2.2.5. Fourier-Transformed Infrared Spectroscopy (FTIR)

The FT-infrared instrument consisted of a Nicolet 380 Fourier-Transformed Infrared Spectroscopy (FTIR) (Thermo Scientific, Madison, WI, USA) and an attenuated total reflectance accessory with a diamond plate (ATR, Smart iTR, Thermo Scientific, Madison, WI, USA). The infrared spectra were collected with Thermo Scientific OMNIC software (version 8.1.11). All spectra were analyzed in the region of 1000–1800 cm^−1^, calculated as a mean of 64 spectra with a resolution of 4 cm^−1^.

#### 2.2.6. Physical Stability Studies

The co-amorphous powders were stored in desiccators under dry conditions (silica gel) at room temperature and at 40 °C, respectively. In order to detect the potential recrystallization of drug and amino acids, samples were analyzed using XRPD at predefined time points: Day 0, 1, 14, 28, 56, 84, 112, 140, 168, 248 and 332.

#### 2.2.7. Dissolution Testing

##### Intrinsic Dissolution Procedure

The intrinsic dissolution rate (IDR) was determined from powder discs compressed using a hydraulic press (Perkin Elmer, Hydraulische Presse Model IXB-102-9, Ueberlingen, Germany). One hundred fifty milligrams of powder containing a co-amorphous mixture or crystalline NAP were compressed at a pressure of 124.9 MPa for 10 s, resulting in a disc with a flat circular surface area of 0.7854 cm^2^, available for contact with the dissolution media. The aluminum cylinder containing the sample was placed in 900 mL of 0.01 M phosphate buffer (pH 7.2, 37 °C) and stirred at a rotation speed of 50 rpm. Aliquots of 5 mL were withdrawn at predetermined time points (2, 6, 10, 15, 20, 25 and 30 min) and immediately replaced with 5 mL of dissolution media. The samples were analyzed the same day using the HPLC method described below. All dissolution experiments were conducted in triplicate.

The IDR of the co-amorphous mixtures was calculated based on the accessible surface area of NAP on the disc by subtracting the area occupied by the amino acids from the total area of the disc using the approach of Allesø *et al*. [11]. Surface calculations were based on mass and densities as given in Table 1, on the assumption that the drug was homogenously distributed throughout the powder mixture and that the accessible area remained constant during the experiment.

##### HPLC Analysis

All samples from the dissolution experiment were analyzed using a Merck Hitachi (Tokyo, Japan) HPLC system consisting of an AAS.200A autosampler, a L-6200A low pressure ternary gradient pump, a LaChrom L-7400 UV detector (VWR-Bie & Berntsen, Soeborg, Denmark) and a Discovery^®^ C18 (10 cm × 2.1 mm, 5 µm) column (Supelco Analytical, Bellefonte, PA, USA) kept at 30 °C with a Shimadzu (Kyoto, Japan) column oven CTO-10AC. An Edaq Powerchrom datasystem (version 2.3.3, eDAQ, Denistone East, New South Wales, Australia) was used for data analysis. The UV detection was carried out at 262 nm. The flow rate was kept constant at 1.0 mL/min. The mobile phases consisted of phosphoric acid (0.02 M, pH 2) and MeOH in a ratio of 53:47 (*v*/*v*) resulting in a retention time of approximately 3.6 min. The chromatograms were analyzed on PowerChrom software (version 2.3.3, eDAQ, Denistone East, New South Wales, Australia). Standard solutions were prepared in concentrations of 20, 15, 10, 5, 0.1 and 0.05 μg/mL by dissolving the drug in 25 mL MeOH and subsequent dilution with 0.01 M phosphate buffer, pH 7.2. The resulting standard curve was linear (*R*^2^ = 0.997).

##### Data Analysis

The slopes of the dissolution curves were compared by applying linear regression with a 95% confidence interval. Hence, the curves were considered different if *p*-values were smaller than 0.05. The dissolution behavior of the various formulations was compared at each time point by performing a single-factor ANOVA, likewise with a 95% confidence interval.

## 3. Results and Discussion

### 3.1. Determination of Successful Amorphization by XRPD

**Figure 1:**
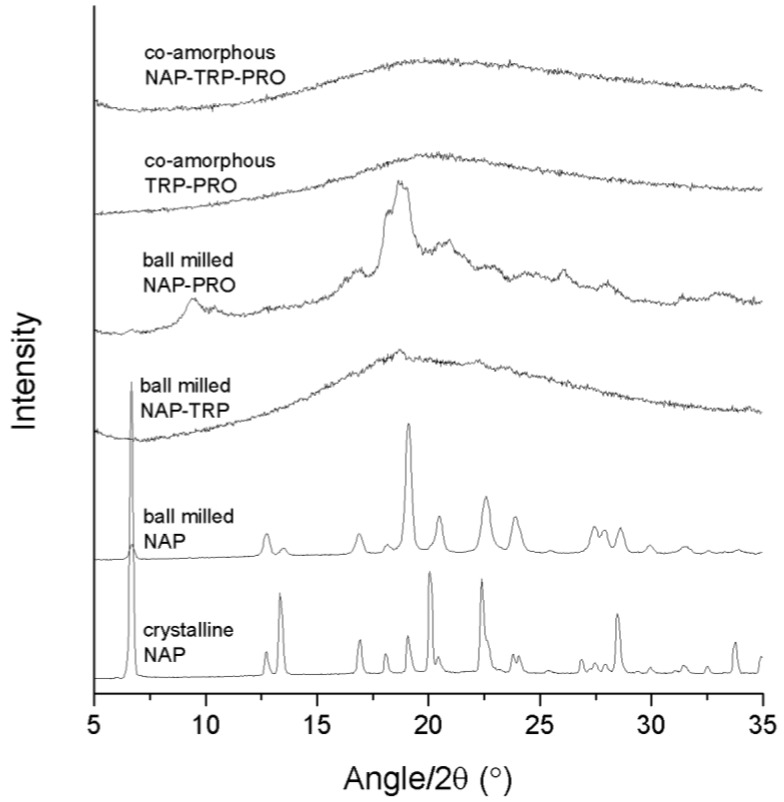
X-ray powder diffraction (XRPD) diffractograms of crystalline NAP, ball-milled NAP, ball-milled NAP–TRP, ball-milled NAP–PRO, co-amorphous TRP–PRO and co-amorphous NAP–TRP–PRO.

Success in amorphization was ascertained by the detection of a halo as the only feature in the XRPD diffractogram. The analysis of the pure compounds revealed that neither NAP (Figure 1) nor any of the amino acids could be transformed into an amorphous solid when ball milled alone for 90 min (data not shown). The preferred orientation of the NAP molecule caused the observed changes in the relative intensity of the X-ray reflections upon milling. In the following, various amino acid and drug combinations with and without PRO were prepared to investigate the effect of adding a highly soluble amino acid to a co-amorphous system. All samples were prepared at a molar ratio of 1:1 (drug:amino acid) or 1:1:1 (drug:amino acid:PRO).

The analysis of the NAP–TRP mixture upon ball milling revealed small remaining reflections in the diffractogram, which can be related to the crystalline form of NAP (Figure 1). It has been shown that process parameters can have a great influence on the reduction in the reflection intensities towards a completely amorphous mixture [10]. However, remaining reflections could still be observed for NAP–TRP after 6 h of ball milling (data not shown). These results are in contrast to previous findings, where the use of TRP was found to be particular advantageous when forming co-amorphous mixtures with a drug [13]. The size and flexibility of the molecule are known to be important characteristics when choosing a good amorphous stabilizer [3,23]. The previous results suggest that TRP tends to have the molecular size and flexibility required to be a good amorphous stabilizer. However interactions between TRP and NAP appear to be insufficient to create a completely co-amorphous mixture at the applied process conditions.

**Figure 2:**
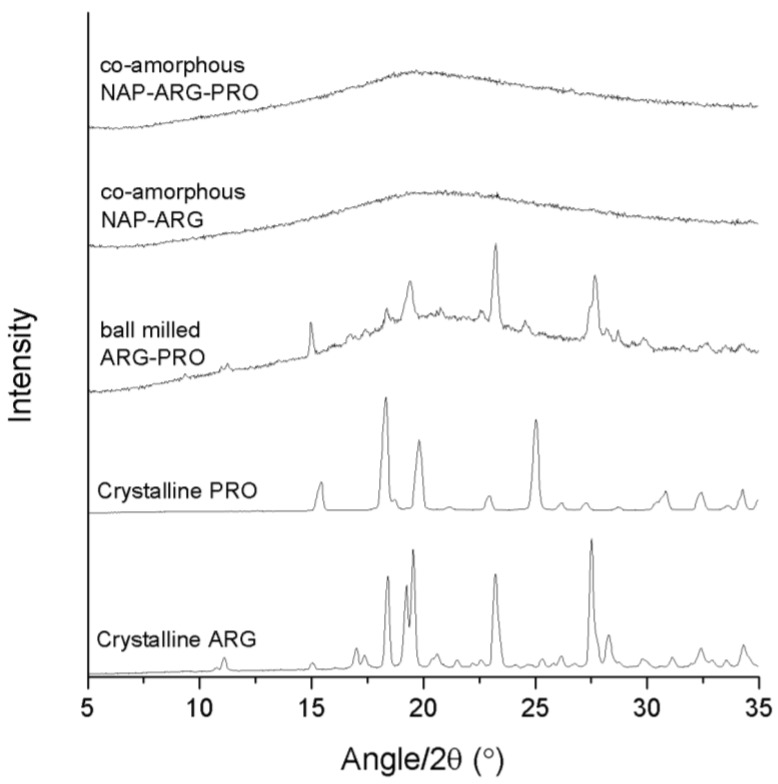
XRPD diffractograms of crystalline ARG, crystalline PRO, ball-milled ARG–PRO, co-amorphous NAP–ARG and co-amorphous NAP–ARG–PRO.

Ball milling of NAP–PRO showed remaining crystallinity in the XRPD diffractogram, which can be related to crystalline NAP and crystalline PRO (see Figure 2). This indicated that PRO alone is not sufficient in stabilizing a co-amorphous system with NAP. Generally, rigid molecules, like PRO, are not easily transformed into their amorphous form, because of their stiff structure and high tendency to arrange in an ordered crystal lattice, in contrast to more flexible molecules [3,23]. Co-milling of TRP–PRO resulted in a co-amorphous mixture, suggesting interactions between the two amino acids (Figure 1). Ball milling of the ternary mixture, NAP–TRP–PRO, resulted in an amorphous blend (Figure 1). Thus, the addition of PRO had a positive effect on the amorphization of NAP, which could not be achieved by any of the amino acids alone.

The binary mixture, NAP–ARG, was found to be amorphous after ball milling, in contrast to an earlier publication, where small crystalline reflections were seen in the X-ray diffractogram upon ball milling of NAP–ARG (Figure 2) [18]. Variation within these results probably is due to differences in milling time and milling frequency. Ball-milled ARG–PRO did not result in a co-amorphous mixture, and the remaining reflections can be correlated to a combination of crystalline ARG and PRO being present in the mixture (Figure 2). The ternary mixture NAP–ARG–PRO was found to be amorphous, as only a halo was detected by XRPD. Summarizing, the presence of ARG stabilized co-amorphous mixtures as observed in co-amorphous NAP–ARG and NAP–ARG–PRO. In the case of NAP–TRP, a small remaining degree of crystallinity was observed, while NAP–TRP–PRO resulted in a co-amorphous mixture.

### 3.2. Determination of Thermal Properties by DSC

**Figure 3:**
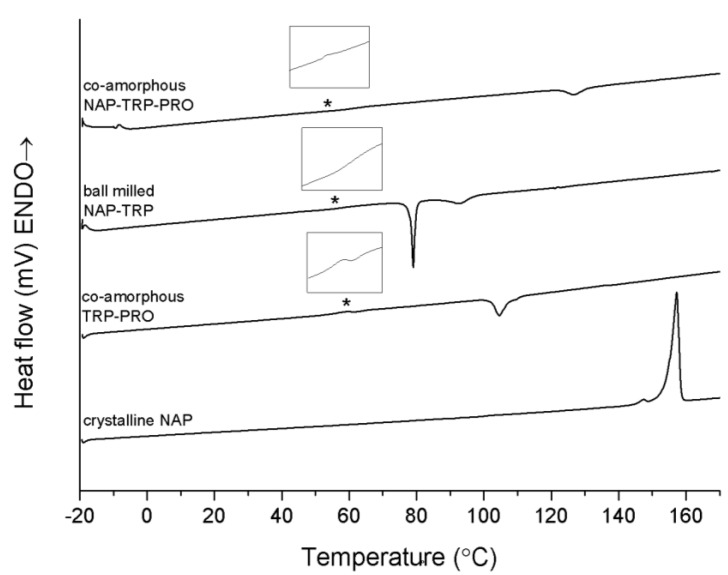
DSC measurements of crystalline NAP, co-amorphous TRP–PRO, ball-milled NAP–TRP and co-amorphous NAP–TRP–PRO. The T_g_ is marked by asterisk (*) and enlarged in the insert above the graph. Endothermal events up.

DSC studies were carried out in order to identify and characterize the thermal properties of the co-amorphous mixtures. In the DSC thermogram of pure crystalline NAP, a large endothermic event at approximately 157 °C was observed, which represented the melt of the crystalline anhydrous state (Figure 3) [15,24,25]. As NAP on its own cannot be transferred into an amorphous form by ball milling, the T_g_ of NAP (5.0 ± 0.4 °C) was determined via *in situ* quench cooling in a DSC using a heating rate of 10 K/min, as previously published [26]. Figure 3 further shows the thermograms of the binary and ternary co-amorphous systems containing TRP. The sample containing NAP–TRP had a T_g_ around 58 °C, followed by a re-crystallization of TRP. No melting of crystalline NAP was observed in the thermogram, although the XRPD diffractogram showed small remaining crystalline NAP reflections in this sample (see Figure 1). A possible explanation could be that NAP and TRP form a homogeneous phase with one T_g_, spiked with small amounts of remaining NAP crystals that can be identified with XRPD after the milling. Upon heating above T_g_, the remaining NAP crystals dissolve within the NAP–TRP blend in its supercooled liquid state. However, this phase is also supersaturated with respect to TRP in the amorphous NAP, leading to TRP partly recrystallizing above the T_g_. This leaves an amorphous NAP phase saturated with TRP, which is stable enough to prevent crystallization of NAP. Thus, no NAP melting endotherm was detected in the NAP–TRP thermogram.

The co-amorphous NAP–TRP–PRO mixture consisted of a homogeneous phase reflected as a single T_g_ in the thermogram. A small exothermic peak could be observed at approximately 120 °C, which is due to the recrystallization of TRP, confirmed by XRPD measurements of the powder after pre-heating above 95 °C (data not shown). Recrystallization of TRP can further be observed in co-amorphous TRP–PRO (Figure 3). The re-crystallization event occurs at a higher temperature and with a lower enthalpy in the ternary mixture compared to NAP–TRP and TRP–PRO, suggesting a more stable and homogeneous mixture, even though the T_g_ of the NAP–TRP–PRO (55.1 ± 3.1 °C) is lower than that of NAP–TRP (58.2 ± 0.5 °C) and TRP–PRO (67.2 ± 6.8 °C). Thus, the presence of both TRP and PRO improves the ability to form a co-amorphous mixture. This is further confirmed by the XRPD measurements presented in Figure 1; showing that complete amorphization of the formulation after 90 min of ball milling requires the presence of both TRP and PRO. However, the DSC results discussed above suggest additional interactions when NAP is added to TRP–PRO.

A benefit of applying TRP–PRO as an amorphous stabilizer in co-amorphous formulations is the high T_g_ (67.2 ± 6.8 °C) of this system compared to the T_g_ for amorphous NAP (5.0 ± 0.4 °C) (Table 2) [13]. According to the Gordon Tayler equation, the glass transition temperature of a co-amorphous system is expected to have a value between the corresponding T_g_s of the components in the blend [22]. Thus, there is a high probability to obtain an increased T_g_ of the co-amorphous mixture consisting of a drug together with TRP–PRO, compared to the amorphous drug alone. A theoretical T_g_ for co-amorphous NAP–TRP–PRO of 37.2 °C is calculated by the Gordon Taylor equation (Table 2), which is considerably higher than for amorphous NAP alone. However, when comparing to the experimental T_g_, an even further increase in T_g_ (55.1 ± 3.1 °C) was observed, suggesting strong molecular interactions between the molecules in the co-amorphous NAP–TRP–PRO mixture, as the Gordon Taylor equation assumes an amorphous blend without interactions [22]. These results indicate TRP–PRO as being beneficial to combine with NAP in order to obtain a thermally-stable co-amorphous system with a high T_g_. However, the T_g_ of amorphous TRP (140.2 ± 1.0 °C) is even higher, which explains why this amino acid previously has contributed to stable co-amorphous systems.

**Figure 4:**
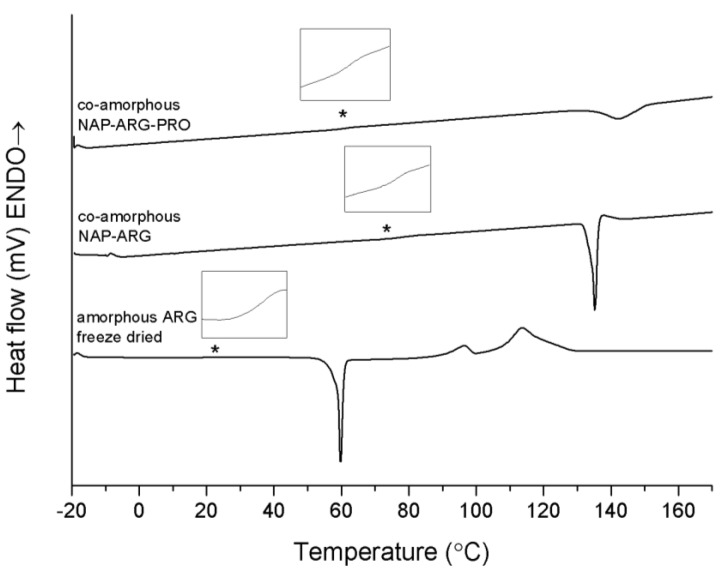
DSC thermograms of amorphous freeze-dried ARG, co-amorphous NAP–ARG and NAP–ARG–PRO. The T_g_ is marked by asterisk (*) and enlarged in the insert above the graph. Endothermal events up.

Co-amorphous NAP–ARG and NAP–ARG–PRO both showed a single T_g_ at 72.1 ± 2.9 °C and 63.5 ± 1.9 °C, respectively, followed by a re-crystallization exotherm (Figure 4). XRPD measurements of the mixtures pre-heated to 160 °C suggest that NAP and ARG crystallize in both mixtures. However, no NAP endotherm can be observed in the thermogram, which indicates that cooling before the XRPD measurements could have caused NAP to crystallize. The crystallization peak observed in the thermogram is expected to be ARG, which appears at a higher temperature and with a lower enthalpy when PRO is added. These results suggest again that PRO contributed to a stabilization of the co-amorphous system, similar to NAP–TRP–PRO.

**Table 2:** Experimental T_g_s of the pure amorphous quench-cooled NAP [26], amorphous freeze-dried ARG, ball-milled TRP and ball-milled co-amorphous blends (*n* = 3, mean ± standard deviation).

Sample Content	T_g_ (°C)	Theoretical T_g_ (°C)
NAP	5.0 (±0.4)	–
ARG	18.4 (±2.7)	–
TRP ^1^	140.2 (±1.0)	–
NAP–ARG	72.1 (±2.9)	10.5
NAP–ARG–PRO	63.5 (±1.9)	8.2
NAP–TRP–PRO	55.1 (±3.1)	37.2
NAP–TRP ^1^	58.2 (±0.5)	54.6
TRP–PRO	67.2 (±6.8)	–
ARG–PRO ^2^	11.0 (±4.1)	–

^1^ small remaining reflections could be observed upon milling of NAP–TRP; however, it was possible to measure a single T_g_; ^2^ Amorphous TRP and ARG–PRO was ball milled for 6 h.

It is obvious from Table 2 that mixtures containing ARG resulted in T_g_ values considerably higher than the theoretical calculations. This can be explained by ionic interactions, due to salt formation, which is not considered in the Gordon Taylor calculation. One of the reasons for the low theoretical calculated T_g_ of the co-amorphous systems is the low T_g_ of amorphous ARG, measured as 18.4 ± 2.7 °C of the freeze-dried substance (Table 2). This T_g_ is lower than previously reported, where T_g_s of 42 °C [27] and 55 °C [28] were found. The low T_g_ of amorphous ARG indicates that salt formation or other strong interactions between the amino acid and other components in the co-amorphous system are required to obtain a co-amorphous system with ARG.

The T_g_ of both ternary systems is lower than those of the corresponding binary mixtures without PRO. This is likely to be a consequence of the low T_g_ of PRO, decreasing the T_g_ of the co-amorphous blend. Unfortunately, this could not be confirmed experimentally, as it was not possible to prepare amorphous PRO.

### 3.3. Investigation of Molecular Interactions by FTIR

A qualitative assessment of molecular interaction in the co-amorphous mixtures, as well as the molecular arrangement was performed, based on vibrational spectroscopy. Ideally, the IR spectra of the co-amorphous samples are compared to the IR spectra of each of the components in the amorphous form, as the spectrum of the amorphous form has a tendency to exhibit reduced signals, peak shifts and slightly broader peaks compared to the corresponding crystalline spectrum [29,30]. As neither NAP nor any of the amino acids were amorphous after 90 min of ball milling, reference spectra were obtained for the amorphous quench-cooled NAP, amorphous freeze-dried ARG and amorphous TRP prepared by 6 h of ball milling. These spectra, as well as the spectra of co-amorphous TRP–PRO and crystalline amino acids were used as the reference. The spectral region between 1000 and 1800 cm^−1^ was chosen for analysis, as it contains information regarding changes in the aromatic ring systems (1100–1500 cm^−1^), as well as hydrogen bonded carboxylic acids (1700 cm^−1^) and amides (1600 cm^−1^) [30].

**Figure 5:**
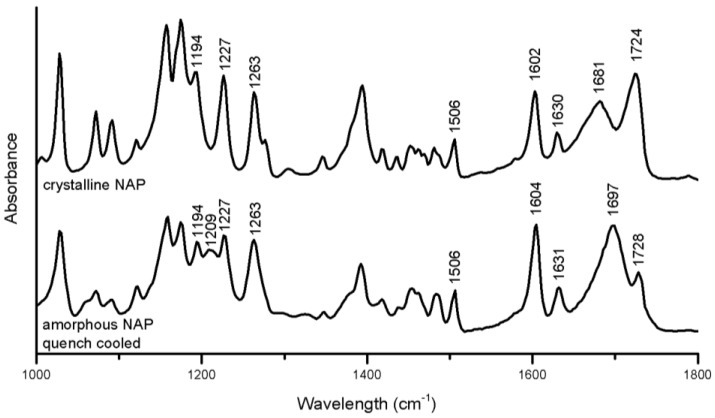
FTIR spectra of crystalline and amorphous NAP prepared by quench cooling.

The crystalline to amorphous transformation of NAP induced the following changes in the spectrum (Figure 5): peak alterations were observed in the aromatic region related to changes in the molecular environment of the naphthalene moiety (1194–1263 cm^−1^) [30] the peak at 1681 cm^−1^ referring to the hydrogen-bonded carbonyl stretch was increased and shifted to 1697 cm^−1^, and the free carboxylic acid group peak at 1724 cm^−1^ was reduced and shifted to 1728 cm^−1^ [31]. Generally, shifts of functional groups upon amorphous to crystalline transformations or in amorphous mixtures indicate changes of intermolecular interactions between the molecules, e.g., a changed hydrogen bonding pattern [14,30]. The changes observed in amorphous NAP suggest the formation of NAP dimers interacting via hydrogen-bonds, including the carboxylic acid groups upon amorphization, as previously reported [30]. The changes in the aromatic region further indicated a change in the π–π interactions.

#### 3.3.1. Interaction in Tryptophan (TRP) Containing Mixtures

**Figure 6:**
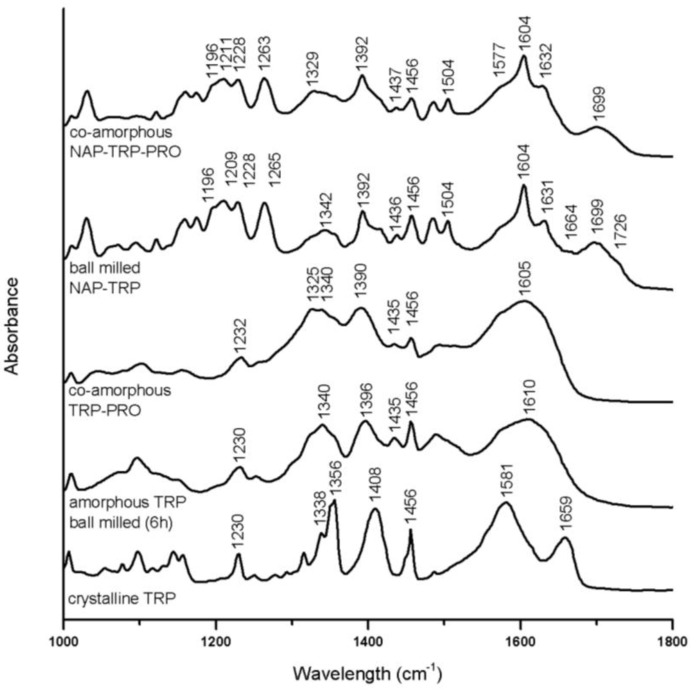
FTIR spectra of crystalline TRP, amorphous TRP ball milled for 6 h, co-amorphous TRP–PRO, the ball-milled mixture, NAP–TRP, and co-amorphous NAP–TRP–PRO.

Several alterations can be observed in the TRP spectra upon amorphization after 6 h of ball milling. Broader bands, as well as decreased peak intensities can be observed for the aromatic region (1200–1500 cm^−1^) (Figure 6) [32]. Besides the general broadening of the peaks, the most pronounced change is related to the CO_2_^−^ asymmetric stretch at 1659 cm^−1^ and the amide band at 1581 cm^−1^ in crystalline TRP, which are combined to one broad band around 1610 cm^−1^ in amorphous TRP [33].

In co-amorphous TRP–PRO, the C=O stretches or amid bands usually found between 1500 cm^−1^ and 1700 cm^−1^ in the crystalline spectra fused to a broad band with a maximum around 1605 cm^−1^, as also observed for amorphous TRP. These findings, as well as a further broadening of the peaks in the aromatic region compared to amorphous TRP are an indication of molecular interactions. Strong interactions between the TRP and PRO molecules comply well with the DSC data, which showed a particularly high T_g_ for the mixture of these amino acids (Table 2).

This combined band together with amorphous NAP peaks is seen in the spectrum of amorphous NAP–TRP as a peak with a maximum at 1604 cm^−1^. The carbonyl symmetric stretch at 1396 cm^−1^ in amorphous TRP is further decreased in NAP–TRP, which indicates that this group is involved in interactions with NAP. The crystalline TRP band at 1659 cm^−1^ is transformed into a peak with decreased intensity and maximum at 1664 cm^−1^ in the NAP–TRP spectrum, which supports the occurrence of interactions involving CO_2_^−^ [33]. The peaks in the aromatic region of the NAP–TRP spectrum are similar to amorphous TRP and can further be compared to the alterations previously observed upon forming co-amorphous carbamazepine–TRP, where hydrogen bonds, including the TRP carboxylic acid and amide group, as well as π–π interactions between the aromatic rings, were observed [14]. The major alterations in the NAP–TRP spectrum related to NAP are an additional reduction and shifts in the hydrogen bonded (1699 cm^−1^) and the free carboxylic acid (1726 cm^−1^) peaks, compared to amorphous NAP. These findings support that NAP–TRP heterodimers are formed upon ball milling, interacting via hydrogen bonds involving the carboxylic group in NAP in contrast to the homodimers in amorphous NAP. Similar heterodimers have been observed between NAP and indomethacin upon amorphization [26,30]. The peak observed at 1726 cm^−1^ in the spectrum for ball-milled NAP–TRP indicates free carboxylic groups, which can be related to unbound NAP molecules, as also observed for crystalline and amorphous NAP (Figure 6). In a completely co-amorphous mixture, all NAP molecules are expected to be involved in interactions. Thus, free C=O groups confirm the presence of crystalline NAP molecules in the mixture, observed as small remaining reflections by the XRPD (see above). Changes in the aromatic region of the spectra upon amorphization of NAP together with TRP are very similar to the spectra for amorphous NAP, further suggesting π–π interactions, as observed as a consequence of molecular changes of TRP.

In the ternary co-amorphous NAP–TRP–PRO mixture, the distinct peaks referring to free acids and carbonyls (1650–1750 cm^−1^) are transformed into a broad peak with a maximum at 1699 cm^−1^. Additional changes are observed in this region of the NAP–TRP–PRO spectrum compared to NAP–TRP, indicating that all three components are involved in interactions, which is in line with the findings of the DSC measurements (Figure 3). The crystalline TRP amide bond at 1581 cm^−1^ appears to remain as a small shoulder at 1577 cm^−1^ in co-amorphous NAP–TRP–PRO. However a similar pattern is observed in the spectra of amorphous TRP and co-amorphous TRP–PRO, suggesting that the shoulder is not an indication of remaining crystallinity (Figure 6). Alterations related to the TRP amide, as well as the aromatic vibrations in NAP and TRP are almost identical in the ternary mixture and ball-milled NAP–TRP, which indicates π–π interactions besides hydrogen bonds in co-amorphous NAP–TRP–PRO.

Overall, the changes in the aromatic region of NAP and TRP indicate π–π interactions in ball-milled NAP–TRP and co-amorphous NAP–TRP–PRO. Additional changes in vibrations indicate hydrogen bonding between the TRP carboxylic acid or amide group and the NAP carboxylic acid group in both mixtures. However, unbound NAP molecules appear in ball-milled NAP–TRP, observed as remaining free carbonyl groups, as this mixture is not completely amorphous. PRO contributed to further hydrogen bonding in co-amorphous NAP–TRP–PRO, observed as additional changes in the bands around 1699 cm^−1^, which could explain the absence of unbound NAP molecules, leading to a completely amorphous mixture.

#### 3.3.2. Salt Formation in Arginine (ARG) Containing Mixtures

**Figure 7:**
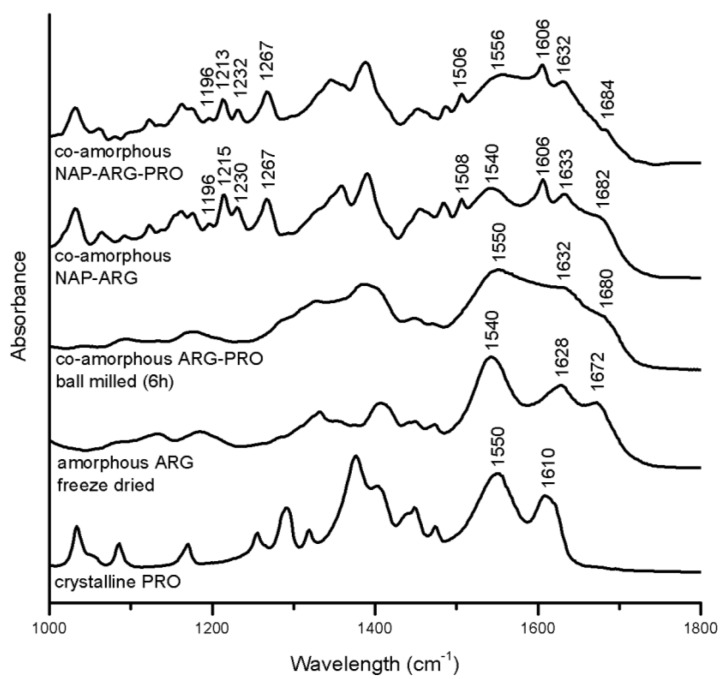
FTIR spectra of crystalline PRO, as well as the spectra of amorphous freeze-dried ARG, co-amorphous ARG–PRO ball milled for 6 h, co-amorphous NAP–ARG and co-amorphous NAP–ARG–PRO.

Several indications for interactions in the ARG containing mixtures are seen with FTIR (Figure 7). In the spectrum of co-amorphous NAP–ARG, the free carboxylic acid band of NAP (1697 and 1728 cm^−^^1^) cannot be observed, indicating a salt formation between the acidic drug and the basic amino acid, as also suggested earlier [18]. The corresponding ionized carboxyl group, which usually appears around 1505–1610 cm^−1^ [34], is difficult to identify, due to overlapping peaks, similar to previously investigated co-amorphous indomethacin–ARG mixtures [14]. Furthermore, the amide vibration at 1540 cm^−1^, as well as vibrations related to the guanidyl group (1600–1700 cm^−1^) are reduced compared to amorphous ARG, supporting the likelihood of salt formation with NAP [32]. Besides the salt formation, it is possible that the guanidinium group in ARG interacts with the aromatic rings in NAP, resulting in π–π interactions, as described between ARG and other aromatic drugs [16].

In the spectrum of co-amorphous NAP–ARG–PRO, the amide vibration of PRO was shifted from 1550 cm^−1^ in the crystalline spectrum to 1556 cm^−1^, similar to the shoulder in co-amorphous ARG–PRO at 1550 cm^−1^ [35]. Furthermore, the C=O band at 1610 cm^−1^ in the spectrum of PRO cannot be found in the co-amorphous blend [32]. In co-amorphous ARG–PRO, this band has changed into a shoulder at 1632 cm^−1^, which cannot be observed in the spectrum of co-amorphous NAP–ARG–PRO, as it is overlapping with NAP vibrations. These changes are indicative of molecular interactions between PRO with either one or two of the other components, NAP and ARG, in the ternary mixture. The alteration in the NAP aromatic region observed in NAP–ARG and NAP–ARG–PRO could be associated with π–π interactions, as could the changes in the aliphatic vibration in PRO (1390–1300 cm^−1^) [32]. Similar interactions between the guanidinium group in ARG and the aromatic rings of other drugs have previously been described [16]. The most pronounced interactions in both co-amorphous mixtures, including NAP and ARG, was the salt formation; however, additional interactions involving PRO are suggested to occur in the ternary mixture.

### 3.4. Storage Stability

Co-amorphous NAP–ARG and NAP–TRP–PRO were stable throughout the period of the stability study (332 days at room temperature and at 40 °C under dry conditions). The samples where considered stable when observing a halo in the XRPD diffractogram. The co-amorphous NAP–ARG–PRO mixture was stable for approximately 84 days on storage at room temperature and 40 °C (Table 3). Interestingly, the powder collapsed, resulting in a hard solid matrix after 84 days of storage. The XRPD diffractogram of the stored sample revealed small reflections, indicating crystalline PRO in the matrix. The glass transition temperature has often been shown to correlate with the stability of an amorphous system [36], and the reduced stability of co-amorphous NAP–ARG–PRO compared to co-amorphous NAP–ARG can be explained by a lowered T_g_. However, this tendency was not shown for the NAP–TRP–PRO blend, even though the T_g_ was similar to the T_g_ of NAP–ARG–PRO. It is known that strong molecular interactions can have a higher impact on the physical stability of a co-amorphous system than a higher T_g_ [11,26]. From the stability data, it is suggested that PRO contributed less to the overall interactions in a mixture with the NAP–ARG salt, compared to stronger interactions between the molecules in co-amorphous NAP–TRP–PRO. Thus, the suitability of PRO in a co-amorphous mixture might depend on its ability to interact with the other components. In the examples of this study, PRO appears to be a good stabilizer in the non-salt NAP–TRP–PRO blend, enhancing the interactions between all three components and, thus, influencing its amorphization behavior during ball milling, recrystallization upon heating in the DSC and stability during storage, as well as its dissolution behavior (see below).

**Table 3:** Stability of co-amorphous NAP–TRP–PRO, NAP–ARG and NAP–ARG–PRO upon storage at room temperature and 40 °C under dry conditions.

Sample Content	Stability at Room Temperature	Stability at 40 °C
NAP–TRP–PRO	>332 days	>332 days
NAP–ARG	>332 days	>332 days
NAP–ARG–PRO	84 days	84 days

### 3.5. Intrinsic Dissolution Testing

**Figure 8:**
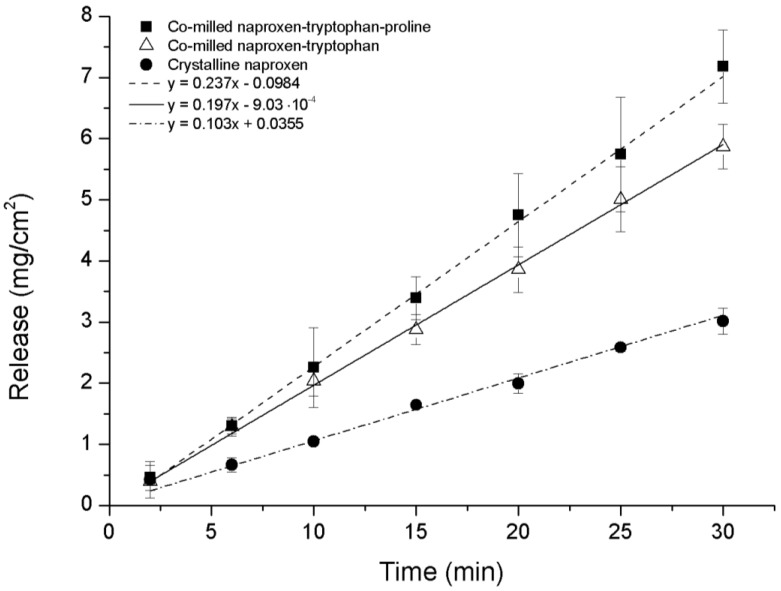
Intrinsic dissolution profiles of crystalline NAP, non-amorphous ball-milled NAP–TRP and co-amorphous NAP–TRP–PRO (*n* = 3, mean ± standard deviation).

The intrinsic dissolution profile of NAP was measured for ternary co-amorphous mixtures containing PRO and binary co-amorphous mixtures and compared to crystalline NAP. Although it was not possible to transform NAP–TRP into a completely co-amorphous mixture, the intrinsic dissolution rate (IDR) of co-milled NAP–TRP was measured and resulted in a 1.9-fold increase compared to crystalline NAP. An even higher dissolution rate of NAP could be found for the ternary co-amorphous systems (Figure 8). Linear regression showed that the curves where significantly different from one another (*p* < 0.05). Further, the amount of NAP dissolved from NAP–TRP–PRO was significantly higher than for NAP–TRP after 15 min and onwards. The improved IDR of NAP can be assigned to the high solubility of PRO combined with the improved stabilization of the co-amorphous system and might be indicative of a three way interaction in the mixture between the components, where the overall gain in the dissolution rate is determined by the solubility of all three components. These findings suggest that it can beneficial to consider the solubility of the amino acid when choosing components for a co-amorphous mixture, resulting in the high dissolution rate of the drug.

**Figure 9:**
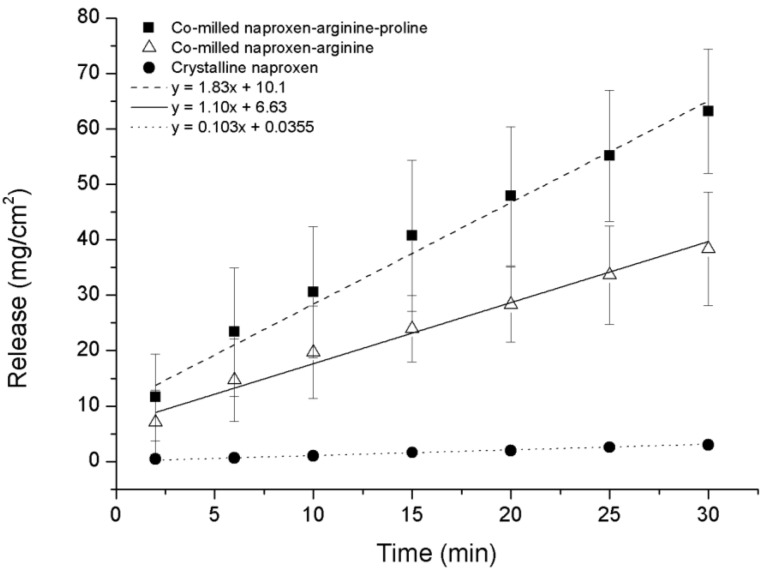
Intrinsic dissolution profiles of crystalline NAP, co-amorphous NAP–ARG and co-amorphous NAP–ARG–PRO (*n* = 3, mean ± standard deviation).

As expected, the IDR of the NAP–ARG salt in the co-amorphous mixture was faster compared to crystalline NAP (Figure 9). A further improvement in the dissolution rate could be observed from the ternary co-amorphous mixture additionally including PRO, resulting in 1.6-fold and 18-fold increases when compared to the NAP–ARG salt and pure crystalline NAP, respectively. Linear regression and ANOVA showed significant improvement in the amount of NAP dissolved from NAP–ARG–PRO observed at all time points, as well as the slope compared to NAP–ARG and crystalline NAP.

In Figure 9, a high *y*-intercept is observed for the dissolution profile of NAP from co-amorphous NAP–ARG, as well as NAP–ARG–PRO systems, indicating a burst release of NAP. A possible explanation could be supersaturation occurring in the area surrounding the disc at the beginning of the dissolution experiment, resulting in the re-crystallization of NAP at the surface of the disc. This theory was supported by XRPD measurements of the disc surface after the dissolution experiment, where crystalline NAP reflections could be observed. Re-crystallization of the drug at the disc surface has previously been observed for other co-amorphous mixtures [11].

The main focus of co-amorphous formulations has previously been to investigate non-salt forming drugs, as alternative methods are needed in order to improve the solubility and dissolution rate of such drugs [13]. Combining the model drug NAP with TRP and PRO confirms the ability of a co-amorphous system to fulfil this formulation purpose by improving the dissolution rate of the drug without forming a salt. However, the high dissolution rate of NAP achieved by combining NAP, ARG and PRO suggested that a co-amorphous system can also further improve the dissolution rate of an amorphous salt. This confirms the potential of the co-amorphous formulation approach, even for salt-forming drug candidates.

## 4. Conclusions

It was possible to prepare stable co-amorphous systems by ball milling that showed molecular interactions between the components of NAP–ARG, NAP–ARG–PRO and NAP–TRP–PRO at a molar ratio of 1:1 and 1:1:1, respectively. The stability of the co-amorphous mixtures could be related to their T_g_ and the degree of molecular interactions between the components in the mixtures. The high T_g_ of amorphous TRP confirmed that this amino acid could be particularly advantageous as a stabilizer in co-amorphous mixtures. However, this was not found for NAP–TRP, which could not be fully converted into a co-amorphous system. The reason for this was shown to be a lack of interactions, as unbound molecules were detectable by FTIR. The neutral co-amorphous system consisting of NAP and TRP was stabilized by the addition of PRO and resulted in an improved IDR compared to the crystalline drug. A larger increase in IDR was found upon forming a co-amorphous salt between NAP and ARG. Although crystalline PRO was observed upon the storage of co-amorphous NAP–ARG–PRO, leading to a reduced stability, the highest IDR was observed for this ternary system. In this study, it was shown that the amino acid, PRO, can improve co-amorphous systems in two ways: firstly, by stabilizing the co-amorphous system and, secondly, by increasing the IDR, with the latter effect being most pronounced when a co-amorphous salt was formed. These results suggest that it is important to consider the solubility of the amino acid and potential interactions between the molecules in order to create at stable co-amorphous system with the additional high dissolution rate of the drug candidate.
